# Male baldness and skin cancer: what are we missing in daily practice?

**DOI:** 10.1590/1806-9282.20241303

**Published:** 2024-12-02

**Affiliations:** 1 Lusiada University Center, Department of Dermatology, São Paulo (SP), Brazil.

It is well-established that continuous exposure to ultraviolet (UV) radiation is highly detrimental to the skin^1,2^. Extensive research has demonstrated the harmful effects of UV light on various skin components, including keratinocytes, fibroblasts, collagen, and blood vessels^1,3^. Over time, the cumulative damage from UV exposure leads to significant alterations in these cellular structures. This ongoing photodamage, combined with the resulting cellular changes, creates an environment that significantly increases the risk of developing skin cancer^1–3^.

As hair restoration procedures have seen exponential growth worldwide due to aesthetic reasons, baldness has been increasingly linked to various other health conditions in the medical literature^4^. For example, depression, anxiety, and even suicidal ideation are frequently reported among bald patients^2,4^. Additionally, baldness has been associated with coronary heart disease, benign prostatic hyperplasia, and prostate cancer^1–3^. However, the clear association between chronic scalp sun exposure and skin cancer is still not commonly addressed in recommendations for bald patients^1^.

A study by Fattah in 2022, involving 271 patients who underwent hair transplantation, revealed a mean age of 35.93±4.40 years. This underscores the increased risk of scalp cancer in bald patients, as UV exposure accumulates over a lifetime^4^. Although there is no precise formula for predicting skin cancer risk, some research suggests that even a relatively modest cumulative UV exposure can significantly contribute to the development of skin cancer^5,6^. For instance, Rosso S. et al. indicated that approximately 2,600 h of unprotected UV exposure might be sufficient to promote the emergence of basal cell carcinomas^7^.

The skincare industry has recognized this issue and has developed products to address it. Modern brands offer hats and caps made from specialized fabrics that filter UV radiation. Additionally, sunscreens designed specifically for men are now available, catering to typically oily skin without leaving a greasy residue^5,6^. Some products also include photoprotection complexes that assist in repairing cellular DNA damage and mitigating actinic damage to some extent. Unfortunately, owing to their high costs, many of these products remain inaccessible to a significant portion of patients, highlighting the need for a more extensive and comprehensive approach^1,7^.

In Brazil, where high sun exposure is common due to cultural practices and frequent outdoor activities, and life expectancy is increasing, scalp cancer remains prevalent despite having over 12,000 board-certified dermatologists^6^. A study by the A.C. Camargo Cancer Center (2008–18) found that scalp melanomas accounted for 14.9% of all melanoma cases and were more likely to be invasive, with greater Breslow thickness, compared to melanomas on the trunk and limbs^6^. Additionally, Marçon CR's study of patients with albinism in São Paulo (2010–17) revealed that 43% of skin cancers, including basal and squamous cell carcinomas, occurred on the head and neck, including the scalp^7^.

By the age of 50 years, approximately 50% of men will experience some degree of baldness^1,6^. Male baldness is limited to individuals with a genetic predisposition and is further accelerated by the effects of testosterone and its metabolites on hair follicles^3,6,7^. Like other chronic genetic disorders, it cannot be completely halted, and even with effective treatment, hair loss will inevitably continue^5,6,8^. Consequently, despite advancements in hair treatments, the inevitable thinning of hair will expose the patient's scalp to UV radiation, making it necessary to implement preventive measures against skin cancer as early as possible^6,8^.

Once awareness of this issue is raised, it becomes easier to establish effective therapeutic approaches for each patient. Some measures involve specific prescriptions aimed at reducing existing photodamage, typically recommended by dermatologists^2,5,9^. These treatments include imiquimod 5% cream, 5-fluorouracil 5% cream, cryotherapy, and photodynamic therapy^9^. On the contrary, general measures that can be recommended by any attending physician include the use of physical barriers made of anti-UV fabrics and applying sunscreen to the bald areas^1,2^.

Finally, it is important for physicians to give this issue the attention it deserves. Baldness is common among men, and with aging, both baldness and skin cancer incidences increase^1–3^. Over time, the unprotected bald scalp is inevitably exposed to UV radiation, which is particularly concerning in tropical countries^6^. Despite the focus on cosmetic and anti-aging products, scalp photoprotection is often less emphasized compared to facial care in dermatological consultations. Dermatologists should remain vigilant about this often-overlooked issue, and general practitioners should consider referring patients with baldness for specialized skin evaluations at least once in their lifetime^1^. Together, these efforts could effectively reduce the incidence of skin cancer on the scalp in susceptible individuals^6,7,9^.
